# Radiograph-based epidemiology of impacted teeth in Sana’a City, Yemen: a multicenter retrospective study

**DOI:** 10.1186/s12903-026-08453-w

**Published:** 2026-05-01

**Authors:** Belal Ahmed Ghithan, Mohammed M Ali, Khalil M. Al-Sabai, Abdulmalik M. Al-Arabi, Shaif M. Al-Wadei, Amal S. Abdulghani, Buthain F. Al-Ariqi, Layla G. Salem, Abeer A. Othman, Bushra A. Al-Lakami

**Affiliations:** Faculty of Dentistry, Al-Hikma University, Sana’a, Yemen

**Keywords:** Impacted teeth, Panoramic radiography, Prevalence, Third molars, Epidemiology, Yemen, Multicenter study

## Abstract

**Background:**

Impacted teeth are a common dental anomaly and may be associated with several radiographic findings, including dental caries, root resorption, and cystic changes. Radiograph-based epidemiological data from Yemen remain limited.

**Objective:**

To assess the radiograph-based prevalence and radiographic characteristics of impacted teeth among patients attending panoramic imaging centers in Sana’a City, Yemen.

**Methods:**

A retrospective cross-sectional study was conducted using 16,575 digital panoramic radiographs collected from 12 panoramic imaging centers in Sana’a City between 2020 and 2022. Radiographs were evaluated for the presence of impacted teeth and analyzed according to age, gender, tooth type, location, angulation, level and depth of impaction, and associated radiographic findings.

**Results:**

Impacted teeth were identified in 2,378 radiographs, yielding a radiograph-level prevalence of 14.3%. The highest frequency was observed among patients aged 20–29 years, with a significantly higher prevalence in females. Third molars accounted for 85.9% of all impacted teeth. Vertical and mesio-angular angulations were the most common patterns, while Level B and partial bony impactions predominated. Radiographic findings suggestive of dental caries and root resorption were frequently observed.

**Conclusion:**

Impacted teeth, particularly third molars, were commonly detected among patients undergoing panoramic imaging in Sana’a City. Panoramic radiography is a useful screening tool for identifying impaction patterns and related radiographic features; however, the findings represent radiograph-based estimates from imaging centers and should not be interpreted as population prevalence. Clinical correlation remains essential for definitive diagnosis and management.

## Introduction

Tooth impaction is a common dental anomaly that occurs when a tooth fails to erupt into its normal functional position within the oral cavity due to physical obstruction, inadequate arch space, or an abnormal eruption path. Impacted teeth may remain asymptomatic for extended periods; however, they are frequently associated with complications such as dental caries, periodontal disease, pericoronitis, root resorption of adjacent teeth, and cystic or tumorous changes [1, 2].

The prevalence and distribution patterns of impacted teeth vary considerably across populations and age groups [3, 4], influenced by genetic, environmental, and developmental factors [3]. Third molars are consistently reported as the most frequently impacted teeth worldwide, followed by maxillary canines and mandibular premolars [1]. Impaction is most commonly detected during late adolescence and early adulthood, corresponding to the eruption period of third molars [5].

Radiographic examination plays a central role in the detection and assessment of impacted teeth. Panoramic radiography is widely used in clinical practice because it provides a comprehensive overview of the maxillofacial region [6, 7], allowing evaluation of tooth position, angulation, depth of impaction, and relationships with adjacent anatomical structures [6]. Despite its inherent limitations, panoramic imaging remains a practical and cost-effective screening modality, particularly in large-scale epidemiological and retrospective studies [8].

In Yemen, data describing the prevalence and radiographic characteristics of impacted teeth remain limited, particularly studies incorporating large, multicenter datasets [9]. Population-specific radiograph-based epidemiological information is essential to support early identification of impaction patterns, inform clinical decision-making, and facilitate comparison with regional and international studies [10].

To our knowledge, few studies in Yemen have evaluated impacted teeth using a large multicenter radiographic dataset exceeding 16,000 panoramic radiographs.

Therefore, the present study aimed to evaluate the radiograph-based prevalence and radiographic characteristics of impacted teeth among patients attending panoramic imaging centers in Sana’a City, Yemen. The study further analyzed impaction patterns according to age, gender, tooth type, anatomical location, angulation, level, depth of impaction, and associated radiographic findings. In addition, a focused subgroup analysis of impacted third molars was performed to provide a detailed characterization of the most commonly affected tooth type.

## Materials and methods

### Study design

This study was designed as a retrospective cross-sectional radiographic study based on the evaluation of digital panoramic radiographs obtained from patients attending selected panoramic imaging centers in Sana’a City, Yemen [11].

### Study setting

The study was conducted in 12 panoramic imaging centers distributed across four major districts of Sana’a City (Maeen, Bani Alharith, Al-Sabeyn, and Shoub). These centers represent the primary referral facilities for panoramic radiographic examinations within the city.

### Study population and sampling

The study population included all patients aged 18 years and older who underwent panoramic radiographic examination at the selected centers between 2020 and 2022. A cluster sampling approach was applied, with imaging centers serving as clusters. All available digital panoramic radiographs meeting the eligibility criteria during the study period were included.

### Inclusion and exclusion criteria

Radiographs were included if they were obtained from patients aged ≥ 18 years, acquired during the study period, originated from the selected centers, and were of acceptable diagnostic quality. Radiographs were excluded if they belonged to patients younger than 18 years, demonstrated inadequate image quality, or were obtained outside the defined centers or study period.

### Radiographic assessment and variables

Radiographic evaluation was performed using panoramic images only. The presence of impacted teeth was defined as teeth that failed to erupt into their normal functional position within the dental arch. For prevalence estimation, the unit of analysis was the panoramic radiograph (patient level), whereby the presence of at least one impacted tooth per radiograph was recorded. Detailed characteristics were analyzed at the tooth level, as multiple impacted teeth could be present within a single radiograph.

Variables assessed included age, gender, residential district, tooth type, anatomical location (maxillary/mandibular; right/left), angulation, level and depth of impaction, and associated radiographic findings.

Tooth angulation was classified according to Winter’s classification (vertical, mesio-angular, disto-angular, horizontal, and inverted). The level of impaction was categorized as Level A, B, or C based on the vertical position of the impacted tooth relative to the adjacent cervical line. The depth of impaction was classified as partial bony impaction (PBI), complete bony impaction (CBI), or soft tissue impaction (STI).

Radiographic findings suggestive of dental caries, root resorption of adjacent teeth, and cystic lesions were recorded based on established panoramic radiographic criteria and considered indicative findings rather than definitive clinical diagnoses.

When multiple impacted teeth or findings were present on a single radiograph, each was recorded separately at the tooth level.

### Examiner calibration

Radiographic assessments were performed independently by two calibrated examiners using standardized evaluation criteria. Examiner calibration was conducted prior to the main analysis on a randomly selected subset of 200 panoramic radiographs to assess inter-examiner reliability.

Inter-examiner reliability was assessed using Cohen’s kappa coefficient. Agreement for the detection of impacted teeth at the radiograph level was excellent (κ = 0.87). For angulation classification at the tooth level, almost perfect agreement was observed (κ = 0.98). Kappa values were interpreted according to Landis and Koch criteria. Statistical calculations were performed using SPSS software (version 26, IBM Corp., Armonk, NY, USA).

Associated radiographic findings, including dental caries, root resorption of adjacent teeth, and cystic lesions, were also recorded as aggregated counts. Given the structure of these data, inter-examiner agreement for associated radiographic findings was assessed descriptively and showed substantial agreement between the two examiners. Any disagreements identified during the calibration process were resolved by consensus.

### Statistical analysis

Statistical analysis was performed using IBM SPSS Statistics software (version 26, IBM Corp., Armonk, NY, USA). Descriptive statistics (frequencies and percentages) were calculated for categorical variables. The chi-square (χ²) test was used to assess associations between categorical variables such as age group and gender with the presence of impacted teeth. A *p*-value < 0.05 was considered statistically significant.

### Ethical considerations

Ethical approval was obtained from the Institutional Review Board of Al-Hikma University, Faculty of Dentistry, Sana’a, Yemen. Official permissions were granted by all participating imaging centers. All radiographs were anonymized prior to analysis, and data confidentiality was strictly maintained.

## Results

A total of 16,575 digital panoramic radiographs were reviewed. Of these, 2,378 radiographs demonstrated the presence of at least one impacted tooth, yielding a radiograph-level prevalence of 14.3%, while 14,197 radiographs (85.7%) showed no evidence of tooth impaction. This prevalence is consistent with other studies conducted globally [1, 2].

Because some patients presented with more than one impacted tooth, analyses were conducted at both the radiograph (patient) level and the tooth level. Radiograph-level analysis was used to estimate prevalence, whereas tooth-level analysis was applied to describe detailed impaction characteristics.

### Distribution by age and gender

A statistically significant association was observed between age group and the presence of impacted teeth (χ² test, *p* < 0.001). The highest frequency of impacted teeth was recorded among patients aged 20–29 years, accounting for 42% of all impacted cases, followed by those aged 30–39 years (33%) [3]. Patients aged 60 years and older represented 2% of the impacted cases.

A significant association was also found between gender and the presence of impacted teeth (*p* < 0.001). Females accounted for 60% of radiographs with impacted teeth, while males represented 40% (Table 1).

**Table 1:** Gender distribution of patients with impacted teeth (radiograph-level analysis)

Sex	Frequency	Percentage	*P*-value
Male	943	40%	< 0.001
Female	1435	60%	< 0.001

### Geographic distribution

Based on residential district, the majority of impacted cases were reported from Maeen district (36%), followed by Bani Alharith (29%), Al-Sabeyn (23%), and Shoub (12%) (Table 2).

**Table 2:** Geographic distribution of patients with impacted teeth by residential district (radiograph-level analysis)

District	Frequency	Percentage
Maeen	856	36%
Bani Alharith	690	29%
Al-Sabeyn	547	23%
Shoub	285	12%

### Tooth type and anatomical location

At the tooth level, third molars constituted the majority of impacted teeth (85.9%), followed by canines (12.2%). Other teeth, including premolars, molars, and incisors, accounted for a small proportion of impacted cases (Table 3).

**Table 3:** Distribution of impacted teeth by tooth type (tooth-level analysis)

Tooth Type	Frequency	Percentage
Central Incisor	4	0.1%
Lateral Incisor	4	0.1%
Canine	604	12.2%
First Premolar	16	0.3%
Second Premolar	45	0.9%
First Molar	1	0.0%
Second Molar	25	0.5%
Third Molar	4252	85.9%

Impacted teeth were distributed across all four quadrants. The highest frequency was observed in the upper left quadrant (27.0%), followed by the lower right (25.2%), lower left (24.9%), and upper right (22.9%) quadrants (Table 4).

**Table 4:** Distribution of impacted teeth by anatomical location (tooth-level analysis)

Site	Frequency	Percentage
Upper Right	1134	22.9%
Upper Left	1335	27.0%
Lower Right	1247	25.2%
Lower Left	1235	24.9%

### Angulation, level, and depth of impaction

Vertical angulation was the most frequently observed pattern (36.5%), followed by mesio-angular (34.5%), disto-angular (15.6%), horizontal (13.0%), and inverted (0.4%) angulations (Table 5).

**Table 5:** Distribution of impacted teeth according to angulation, level, and depth (tooth-level analysis)

Angulation	Frequency	Percentage
Inverted	20	0.4%
Horizontal	645	13.0%
Vertical	1809	36.5%
Disto-angular	771	15.6%
Mesio-angular	1706	34.5%

Regarding the level of impaction, Level B was the most common (44.7%), followed by Level C (32.6%) and Level A (22.8%). Partial bony impaction was the predominant depth classification, followed by complete bony impaction and soft tissue impaction.

### Associated radiographic findings

At the tooth level, radiographic findings suggestive of dental caries were identified in 50.8% of impacted teeth, while signs suggestive of root resorption of adjacent teeth were observed in 44.2%. Cystic lesions were detected in 4.2% of impacted teeth (Table 6).

**Table 6:** Radiographic findings associated with impacted teeth (tooth-level analysis)

Pathology	Frequency	Percentage
Dental Caries	2413	50.8%
Root Resorption	2092	44.2%
Cyst	198	4.2%

### Subgroup analysis of impacted third molars

Impacted third molars accounted for 85.9% of all impacted teeth. These were more frequently observed in the mandible than in the maxilla, with a slightly higher distribution on the left side. Vertical and mesio-angular angulations represented the most common impaction patterns among third molars.

Level B was the most frequently observed level of impaction among third molars, followed by Level C and Level A. Partial bony impaction was the most common depth classification.

Radiographic findings suggestive of dental caries and root resorption were frequently associated with impacted third molars.

Percentages are calculated based on the total number of radiographs with at least one impacted tooth (radiograph-level analysis). The gender distribution presented in Table 1 reflects a statistically significant difference in the radiograph-level prevalence of impacted teeth between males and females.

Percentages are based on the total number of impacted radiographs.

Percentages are calculated based on the total number of impacted teeth (tooth-level analysis).

Analysis was performed at the tooth level. Percentages represent the distribution of impacted teeth by anatomical location.

All variables were analyzed at the tooth level. Percentages are based on the total number of impacted teeth.

Radiographic findings are based on panoramic appearance only and represent suggestive radiographic indicators rather than definitive clinical diagnoses. Percentages are calculated at the tooth level.

## Discussion

The present study provides a large-scale, radiograph-based, multicenter descriptive epidemiological profile of impacted teeth among patients attending panoramic imaging centers in Sana’a City, Yemen. Using a substantial dataset of digital panoramic radiographs, this study contributes region-specific data from an understudied population and allows comparison with previously published radiographic studies.

The radiograph-level prevalence of impacted teeth observed in this study (14.3%) falls within the range reported in other radiographic investigations conducted in different populations [8]. Variations in prevalence across studies may be attributed to differences in study design, age criteria, imaging modality, and population characteristics [1]. Because the present analysis was based on panoramic radiographs obtained from imaging centers, the findings represent radiograph-based estimates rather than true population prevalence.

The highest frequency of impacted teeth was observed among young adults aged 20–29 years, consistent with previous studies reporting that tooth impaction is most commonly identified during late adolescence and early adulthood, corresponding to the eruption period of third molars. The lower frequency observed in older age groups may reflect prior extraction of impacted teeth or late spontaneous eruption.

A higher prevalence of impacted teeth was observed among females, which aligns with findings reported in several previous studies [10]. This pattern has been attributed to earlier completion of jaw growth in females, potentially resulting in reduced space for tooth eruption, particularly for third molars [5]. However, other studies have reported no significant gender difference, suggesting that this association may vary across populations.

Consistent with the existing literature, third molars were the most frequently impacted teeth in the present study [1, 9]. The subgroup analysis of impacted third molars provided additional insight into impaction patterns, demonstrating predominance of vertical and mesio-angular angulations and a high frequency of Level B positioning. These findings are comparable to those reported in radiographic studies conducted in different geographic regions.

The predominance of partial bony impaction observed in this study is in agreement with previous reports indicating that partially erupted impacted teeth are commonly detected on panoramic radiographs and may be associated with a higher risk of local complications. Maxillary canines were the second most frequently impacted teeth, reflecting their complex eruption pathway and susceptibility to impaction, as documented in earlier studies.

Radiographic findings suggestive of dental caries and root resorption were frequently associated with impacted teeth, particularly third molars. These findings are consistent with previous studies and may be related to plaque accumulation, difficulty in maintaining oral hygiene around partially erupted teeth, and close proximity to adjacent tooth roots. Cystic lesions were detected less frequently, which is consistent with reports indicating that cyst formation is relatively uncommon compared with other impaction-related findings.

The findings of this study may assist clinicians in identifying high-risk groups for tooth impaction and may support early radiographic screening strategies among young adults.

Panoramic radiography enabled assessment of impacted teeth and associated radiographic features across a large sample size. Although panoramic imaging has known limitations in detecting early caries and subtle root resorption, it remains a widely used and cost-effective screening tool in epidemiological and retrospective studies. Therefore, radiographic findings identified in the present study should be interpreted as indicative rather than definitive clinical diagnoses.

The strengths of this study include its large sample size, multicenter design, and standardized radiographic assessment. These features enhance the internal consistency of the findings and support their relevance for understanding impaction patterns among patients undergoing panoramic imaging. Nevertheless, the retrospective design, center-based sampling, and reliance on panoramic radiographs without routine clinical or CBCT correlation limit causal inference and generalizability.

## Conclusion

Within the limitations of this retrospective, radiograph-based, multicenter study, impacted teeth were commonly detected among patients attending panoramic imaging centers in Sana’a City, Yemen. Young adults and females represented the most frequently affected groups, and third molars accounted for the majority of impacted teeth. Vertical and mesio-angular angulations, Level B positioning, and partial bony impaction were the predominant patterns observed.

Panoramic radiography proved to be a useful screening modality for identifying impacted teeth and their radiographic characteristics [6]. However, clinical examination and supplementary imaging remain essential for definitive diagnosis and treatment planning. The findings represent radiograph-based estimates from imaging centers and should not be interpreted as true population prevalence.

## Limitations

This study is limited by its retrospective design, center-based sampling, and reliance on panoramic radiographs without routine clinical or cone-beam computed tomography correlation. Radiographic identification of certain conditions, such as early dental caries, subtle root resorption, or small cystic lesions, may be imprecise on panoramic images. Additionally, the absence of detailed clinical variables and multivariable statistical analysis restricts causal inference and generalizability of the findings.

Additionally, the absence of multivariable regression analysis limits the ability to control for potential confounding factors.

## Recommendations

1. Early Detection and Monitoring: Routine panoramic radiographic evaluation may be considered for late adolescents and young adults when clinically indicated to, facilitate early detection of impacted teeth and reduce the risk of associated complications.
2. Clinical and Radiographic Correlation: Radiographic findings of impacted teeth should be supported by thorough clinical examination and, when indicated, supplementary imaging modalities such as intraoral radiographs or cone-beam computed tomography (CBCT).
3. Public Health Awareness: Oral health education programs should be implemented to increase public awareness regarding the potential complications of untreated impacted teeth and the importance of timely dental evaluation.
4. Future Research: Prospective longitudinal studies incorporating clinical assessment, inferential statistical analysis, and advanced imaging techniques are recommended to better understand the outcomes and management of impacted teeth in the Yemeni population.

## Data Availability

The datasets generated and/or analyzed during the current study are available from the corresponding author upon reasonable request.

## Abbreviations

PBI: Partial Bony Impaction
CBI: Complete Bony Impaction
STI: Soft Tissue Impaction
IRB: Institutional Review Board
